# Contrast Agents for Hepatocellular Carcinoma Imaging: Value and Progression

**DOI:** 10.3389/fonc.2022.921667

**Published:** 2022-06-02

**Authors:** Ying Zhang, Kazushi Numata, Yuewu Du, Shin Maeda

**Affiliations:** ^1^ Department of Medical Ultrasound, Ningbo Medical Centre Li Huili Hospital, Ningbo, China; ^2^ Gastroenterological Center, Yokohama City University Medical Center, Yokohama, Japan; ^3^ Department of Gastroenterology, Graduate School of Medicine, Yokohama City University, Yokohama, Japan

**Keywords:** ultrasound, MRI, CECT, hepatocellular carcinoma (HCC), contrast media (CM)

## Abstract

Hepatocellular carcinoma (HCC) has the third-highest incidence in cancers and has become one of the leading threats to cancer death. With the research on the etiological reasons for cirrhosis and HCC, early diagnosis has been placed great hope to form a favorable prognosis. Non-invasive medical imaging, including the associated contrast media (CM)-based enhancement scan, is taking charge of early diagnosis as mainstream. Meanwhile, it is notable that various CM with different advantages are playing an important role in the different imaging modalities, or even combined modalities. For both physicians and radiologists, it is necessary to know more about the proper imaging approach, along with the characteristic CM, for HCC diagnosis and treatment. Therefore, a summarized navigating map of CM commonly used in the clinic, along with ongoing work of agent research and potential seeded agents in the future, could be a needed practicable aid for HCC diagnosis and prognosis.

## Introduction

Hepatocellular carcinoma (HCC) has the third-highest incidence in cancers, along with the fourth leading cause of cancer death in 2020 globally. Moreover, cirrhosis, a major source of HCC, composed 2.4% of death with all causes in 2019 according to the WHO. Meanwhile, hepatitis B virus (HBV) and hepatitis C virus (HCV) infection, alcohol abuse, and non-alcoholic steatohepatitis (NASH) are dominating etiological reasons for cirrhosis and HCC. Modern medicine believes the small HCC is preventable and curable through early diagnosis and timely etiological treatment if screening and surveillance could be well conducted for cirrhosis (1). Therefore, non-invasive medical imaging techniques, such as MRI, ultrasound (US), and CT, have contributed to HCC patients’ management (2–6).

For early diagnosis, treatment assessment, and follow-up, multiple medical imaging modalities were improved and adapted in every corner of HCC prevention and supervision. In the past decades, the diagnostic efficacy of medical imaging has been elevated through the improvement of imaging resolution and associated intravenous contrast agents. US elastography and MR elastography are recommended to supervise and assess hepatic fibrosis, which may gradually progress to cirrhosis without medical intervention (7). On the other hand, taking characteristic advantage of the dual blood supply of the liver, transvenous contrast agents depict the liver lesion by illustrating the tumorous blood supply with characteristics of arterial enhancement (wash-in) and portal hypodensity or hyposignal (wash-out). The classical imaging findings of wash-in and wash-out were believed to have a sensitivity of approximately 60% and a specificity of 96%–100% for small HCCs with a size of 10–20 mm. Still, a biopsy is needed in 40% of these lesions. Along with a deeper investigation of clinical research, an experienced radiologist can achieve a much more satisfying diagnostic efficacy through guidelines like the American College of Radiology Liver Imaging Reporting and Data System (ACR LIRADS) (8, 9). As a result, contrast enhancement imaging, like dynamic MRI and contrast-enhanced CT (CECT), is recommended in mainstream guidelines for preoperative HCC diagnosis with certainty. Screening using the non-enhanced US is also recommended for patients at a higher risk of HCC every 6 months. When it comes to contrast-enhanced US (CEUS), though it is not recommended by the World Federation for Ultrasound in Medicine and Biology (WFUMB) guidelines for liver lesion detection due to the narrow window for arterial phase observation (10), some meta-analyses indicated it to be a promising diagnostic approach for HCC with a sensitivity of 93% (95% CI: 91%–95%) and a specificity of 90% (95% CI: 88%–92%) (11), as well as the diagnostic efficacy of 93% in small HCCs (≦2 cm) (12).

Contrast-enhanced imaging for the tumor is a tracer technique of contrast media (CM) in essence. The distribution and dynamic phases of the agent are analyzed for lesion detection and characterization for early diagnosis and possible prognosis prediction. Therefore, a summarized navigating map of CM commonly used in the clinic, along with ongoing work of agent research and potential seeded agents in the future, could be a needed reference work for both physicians and radiologists.

## Blood Pool Contrast Agents

### Ultrasound Contrast Agents

As early as the late 1960s, people found that the microbubbles (MBs) that provide many reflecting interfaces for echo are a good intravascular flow tracer for US imaging (13), and the hydrogen peroxide solution was launched for echocardiography thereafter. According to the inner gas of the MB, US contrast agent (UCA) could be classified into two generations. Air core with the polymeric coat is the so-called first-generation UCA, such as Levovist (Schering, Berlin-Wedding, Germany). The first-generation UCA is a milestone in the history of medical US imaging development, though it comes with defects like unstableness and unsafety (13). Thereafter, inert gas that is enveloped with a lipid shell at a diameter of approximately several micrometers is developed as the second-generation UCA, which is slightly smaller than that of the red blood cell. Taking advantage of materials science and technology development, the second-generation UCA with greater stability and biosafety can achieve a promising diagnostic efficacy for HCC (11, 12), along with the negligible report of anaphylaxis compared with CT and MRI, which means that UCA can be employed for the patients having iodine allergy, chronic kidney disease, hepatic function failure, asthma, and so on. Moreover, the bedside operation with a portable US machine could be performed in the emergency department (ED) and intensive care unit (ICU) as needed. However, concerning clinical practice, CEUS is not good at imaging the hepatic lesion located near the lung and behind the costal bone, due to the so-called shadow zone caused by the costal bone and lung. The other weakness is US attenuation in far-field of a fatty liver can lead to the indefinable hepatic situation.

Currently, sulfur hexafluoride (i.e., SonoVue, Bracco Imaging, Milan, Italy) is the most consumed in the global UCA market, followed by perfluorinated butane (i.e., Sonazoid, GE Healthcare, Oslo, Norway). The former is a pure blood pool agent, while the latter behaves similarly at the beginning but permeates into extravascular space soon after administration, which will be discussed in Section 3.

### Iodinated Agents for Contrast-Enhanced CT

**Figure 1:**
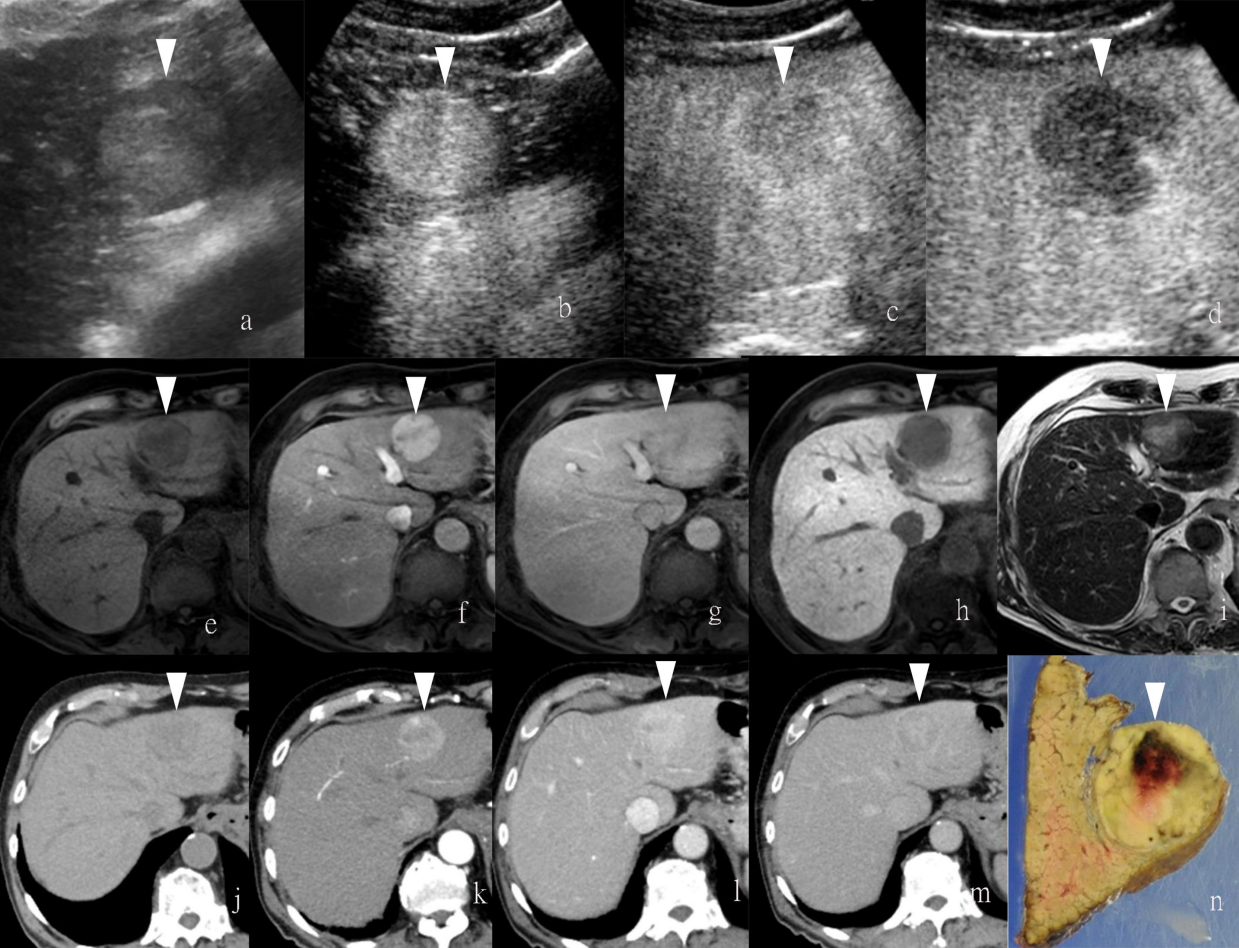
Images of a man in his eighties with a pathological diagnosis of moderately differentiated hepatocellular carcinoma (HCC) and had a history of hepatitis **(C)** At the Sonazoid-enhanced ultrasound (US), the liver lesion at a size of 43 mm with a thin halo located at segment III was observed on B-mode US **(A)**. It was rapidly enhanced in the arterial phase (wash-in) **(B)**, started to fade (wash-out) in portal phase **(C)**, and was totally exhausted in the post-vascular phase **(D)**. At Gd-EOB-DTPA-enhanced MRI, the lesion was hypointense on T1-weighted image **(E)**, with the typical characteristics of wash-in and wash-out from arterial phase, portal phase, to delayed phase **(F–H)**. It showed hyperintensity on T2-weighted image **(I)**. At iodine agent-enhanced CT, it has low-density before enhancement **(J)**. It also showed wash-in and wash-out from arterial phase, portal phase, to delayed phase **(K–M)**. Finally, the gross specimen vividly reflected the morphological information of tumor **(N)**. Arrowheads indicate the margin of the HCC lesion.

Many iodinated agents are pure blood pool agents, which are the widest and longest used CM for X-ray-based enhancement scans (i.e., CECT) (**Figure 1**). To date, the effort of optimizing small-molecule iodinated agents for contrast enhancement could be mainly classified into three eras, including four categories of compounds, from ionic to non-ionic, from monomers to dimers, from high-osmolality to iso-/low-osmolality, associating with decreasing toxicity and increasing bio-tolerability. Commercially available agents are abundant in the clinic, such as iohexol (Omnipaque, GE Healthcare), iopromide (Ultravist, Bayer Healthcare, Leverkusen, Germany), iodixanol (Visipaque, GE Healthcare), iopamidol (Isovue, Bracco Imaging, Milan, Italy), and iothalamate (Cysto-Conray II, Mallinckrodt Imaging, St. Louis, MO, USA). Moreover, novel agents, like iosimenol and GE-145, are on the way to commercialization with the improvements made on an existing basis. The diagnostic efficacy of CECT for HCC in terms of area under the receiver operating characteristic (ROC) curve (AUC), sensitivity, and specificity were reported to be 0.93, 93%, and 82%, respectively (14). For HCC patients, the most distinctive role that CT perfusion imaging has played is the transarterial chemoembolization (TACE) assessment (15). However, despite great improvements that have been made in the bone and cartilage tissue, iodinated contrast agents employed in parenchymal organs, like the liver, have not yet been largely renovated (16, 17).

The blood pool agent applied to MRI is mainly established for MR angiography rather than the liver tumor, which is beyond the scope of the present review article and will not be discussed herein.

## Extracellular Contrast Agents

### Non-Specific Agents

For MRI, gadolinium-based micromolecule agents that have five or seven unpaired electrons could be stimulated to be paramagnetic under an external magnetic field. Those so-called paramagnetic contrast agents for dynamic MRI are developed and enriched (18). Gadolinium chelates (Gd-chelates) are clinically available mainstream for dynamic MRI on T1-weighted images, including Gd-DTPA (gadopentetic acid, Magnevist, Berlex, Berlin, Germany), Gd-DTPA-BMA (gadodiamide, Omniscan, Nycomed Amersham, Amersham, UK), Gd-HP-DO3A (gadoteridol, ProHance, Bracco Diagnostics, Milan, Italy), Gd-DTPA-BMEA (gadoversetamide, Optimark, Mallinckrodt, Staines-upon-Thames, UK), Gd-DOTA (gadoterate, meglumine, Dotarem Guerbet, Princeton, NJ, USA), and Gd-BT-DO3A (gadobutrol Gadovist, Schering Diagnostics, Berlin, Germany). These extracellular agents for non-specific liver MRI are commonly used worldwide because of the good patient tolerance and satisfying diagnostic efficacy (19). Thus, clinical recommendations from guidelines are almost based on the Gd-chelates (8, 9). Moreover, the informative images provided by contrast-enhanced MRI (CEMRI) also contribute to the therapy assessment (**Table 1**).

**Table 1:** The categories of extracellular contrast agents in clinical practice.

Category	Specificity	Class	Classical agents	Featured purposes	Modality
Extracellular agent	Non-specific	Gadolinium chelates	Gadopentetic acid (Gd-DTPA)	Tumor imaging; blood pool imaging	T1 agent for MRI
Reticuloendothelial system (RES) agent (Kupffer cells included)	RES specific	Iron oxide	Ferucarbotran (Feridex)	Liver tumor imaging	T2 agent for MRI
		Microbubbles	Perfluorinated butane (Sonazoid)	Liver tumor imaging; blood pool imaging	Ultrasound contrast agent
Hepatobiliary agent	Hepatobiliary specific	Manganese-based compound	Mangafodipir (Mn-DPDP)	MR cholangiography; liver function indicator	T1 agent for MRI
			Gadobenate dimeglumine (Gd-BOPTA); gadoxetic acid (Gd-EOB-DTPA)	Liver tumor imaging	T1 agent for MRI

### Reticuloendothelial System Endocytosis

Ferumoxytol, a kind of iron oxide nanoparticles (IONPs) approved by the Food and Drug Administration (FDA) as medicine for iron deficiency in adults, was recently reported to be feasible for MR angiography thanks to the characteristic of longer half-life in circulation and the advantage of superparamagnetism (20–23). The so-called negative contrast agents, containing iron oxide particles, darken the normal liver background on T2-weighted images to negatively enhance the target issue, in contrast with the so-called positive agents that brighten the target tissue on T1-weighted images, like Gd-chelates. The first commercially available reticuloendothelial system (RES)-specific contrast agent is ferumoxides (Feridex) (24), which makes lesions that contain negligible RES cells conspicuous on T2-weighted images since the normal liver background containing many RES cells can selectively take up iron oxide particulates to lower the T2 signal intensity (25). Iron oxide crystals coated with dextran or carboxydextran are named superparamagnetic iron oxide (SPIO), which is normally employed as T2 MR CM. With a sufficient infusion of SPIO, normal hepatocytes containing many Kupffer cells are supposed to catch most SPIO particles, leading to a dark area on T2-weighted images. By contrast, tumors, whether benign or malignant, primary or metastatic, that are deficient in Kupffer cells cannot exhibit SPIO uptake, shaping a relatively hyperintense area. However, focal nodular hyperplasia (FNH) seems to be an exception, since SPIO particles may accumulate there and lead to a resultant isointense or even hypointense appearance (26, 27). Following SPIO, the derivative in terms of ultrasmall particulate iron oxides (USPIO) with advantages of convenient administration and striking prolonged plasma half-life that enables it also as a blood pool agent was developed thereafter (28, 29) (**Table 1**).

**Figure 2:**
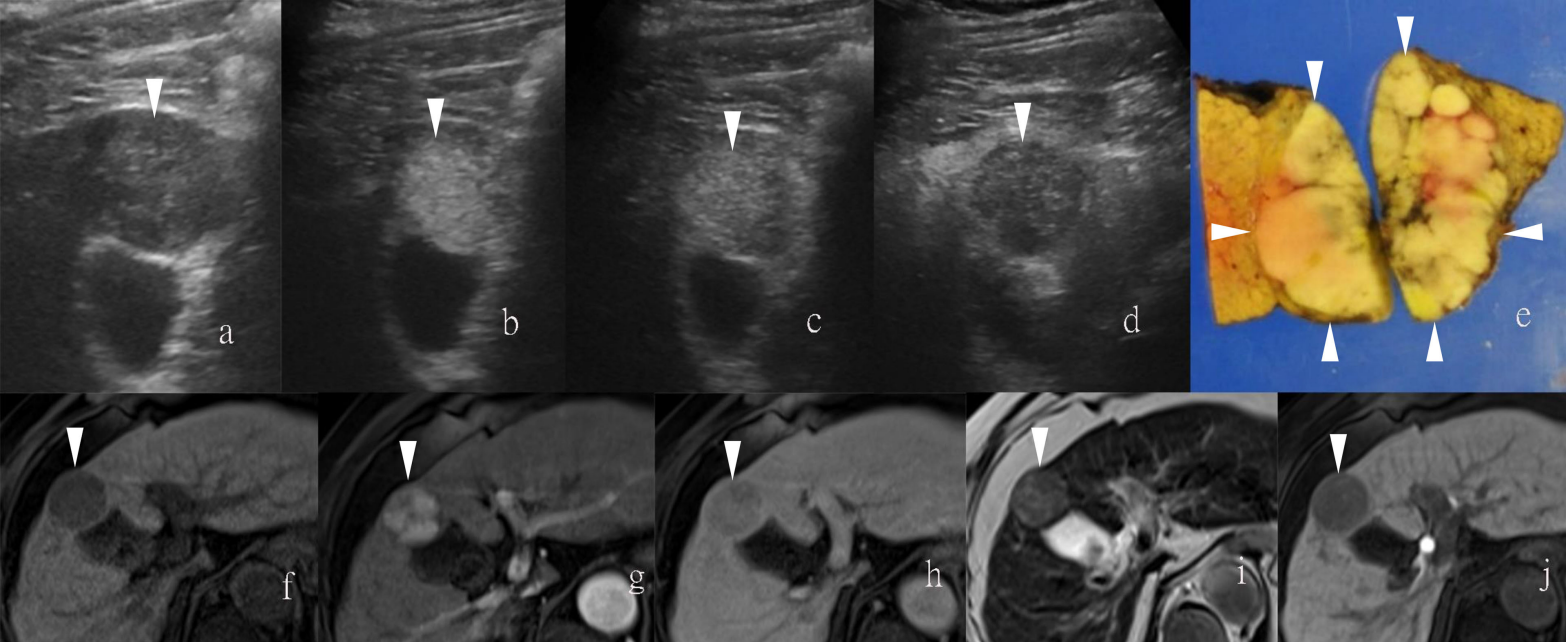
Images of a man in his sixties with a pathological diagnosis of poorly to moderately differentiated hepatocellular carcinoma (HCC) and had a history of cirrhosis. At the Sonazoid-enhanced ultrasound (US), the liver lesion was heterogeneous hyperechoic with the indistinct margin on B-mode US **(A)**. It was rapidly enhanced in the arterial phase (wash-in) **(B)**, still iso-echoic in portal phase **(C)**, and was totally exhausted in the post-vascular phase **(D)**. At Gd-EOB-DTPA-enhanced MRI, the lesion was hypointense on T1-weighted image **(F)**, with uncharacteristic wash-in and delayed wash-out from arterial phase to delayed phase **(G, H)**. It showed hyperintensity on T2-weighted image **(I)**. The contrast media (CM) were totally exhausted till the hepatobiliary phase **(J)**. The gross specimen indicated the heterogeneous pathological differentiation of HCC **(E)**. Arrowheads indicate the margin of the HCC lesion.

**Figure 3:**
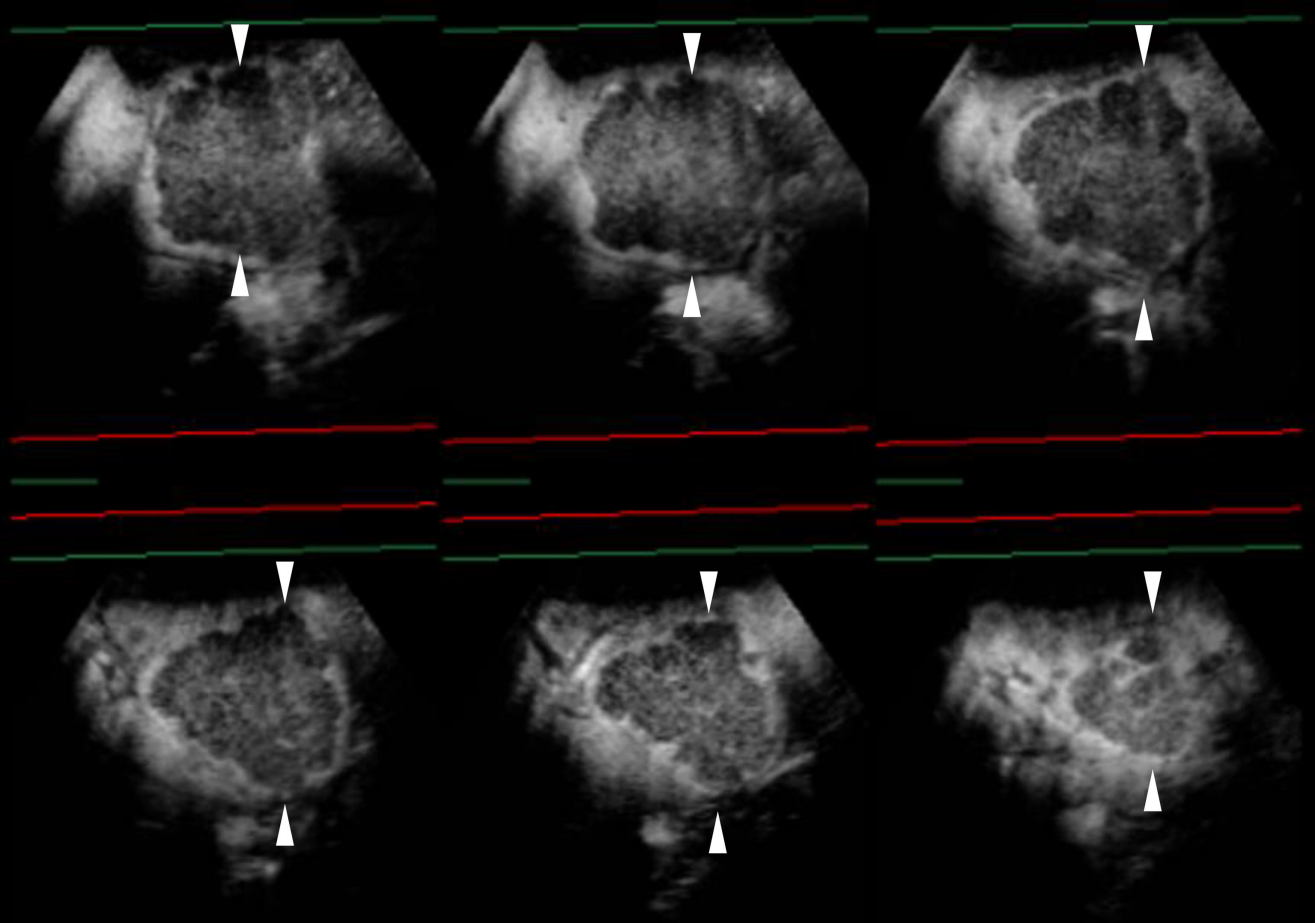
Sonazoid-enhanced ultrasound (US) images of a man in his seventies with a pathological diagnosis of moderately differentiated hepatocellular carcinoma (HCC), who had a history of hepatitis C. The tumor was 70mm. Consecutive lateral images of the tumor remarkably illumed the irregular margin on the three-dimensional (3D) US, which was obtained by auto-sweep 3D scanning in the post-vascular phase. Tomographic ultrasound images in plane A, which can be translated from front to rear, with a slice distance of 4.8 mm. Arrowheads indicate the margin of the HCC lesion.

**Figure 4:**
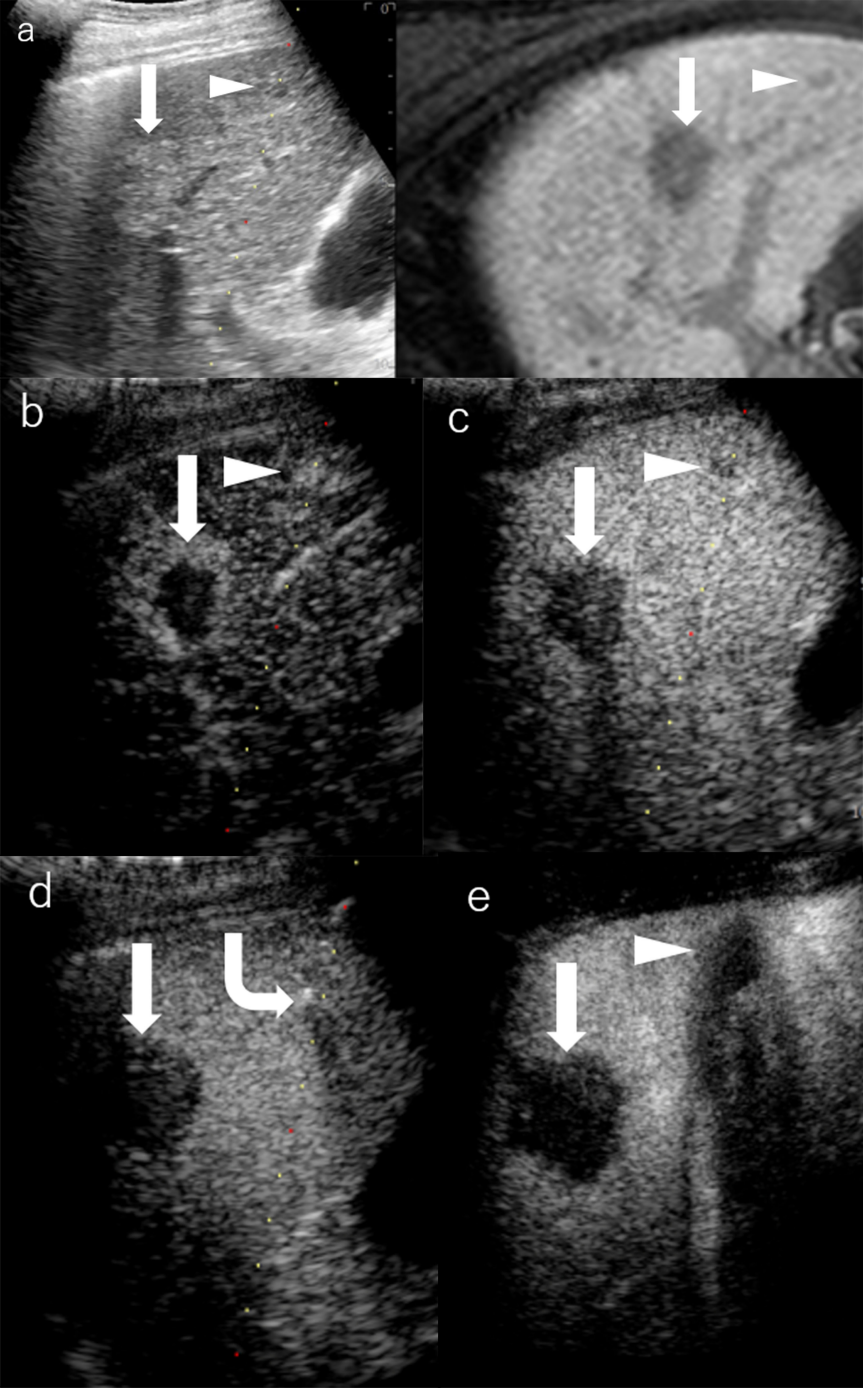
Images of a man in his seventies with a pathological diagnosis of moderately differentiated hepatocellular carcinoma (HCC) and had a history of cirrhosis and HCC. The hepatobiliary phase of EOBMRI (right side), as the reference, was combined with conventional grayscale US (left side), displayed an 8-mm indistinctive hypointense area (the triangular arrow) in segment V on the same screen for the fusion imaging **(A)**. The extrasmall lesion was hypervascular in the arterial phase of Sonazoid-enhanced ultrasound (US) **(B)**, while the post-vascular phase indicated it to be a slightly hypoechoic area **(C)**. Pathway guidance was ready for radiofrequency ablation (RFA) needle manipulation on real-time US **(B–D)**, along with tracking for the metallic needle tip (the curved arrow) **(D)**. The contrast-enhanced US (CEUS) evaluated the target ablation area to be non-enhanced after RFA **(E)**. Arrows indicate the margin of the bigger HCC lesion, which was previously treated by RFA. And Arrowheads indicate the margin of the extrasmall HCC lesion.

Regarding UCA, Sonazoid is an MB of perfluorobutane core wrapped by the shell of hydrogenated egg phosphatidylserine. At first, Sonazoid MBs were used as the blood pool contrast agent. As early as 1 min after the intravenous administration, the MBs start to diffuse into extravascular and intercellular space where they will be phagocytosed by the Kupffer cells in the normal liver sinusoids. Approximately 10 min later, once intravascular MBs are mostly eliminated, the remaining stable MBs endocytosed by resident macrophages in liver parenchyma will shape the so-called additional Kupffer phase or post-vascular phase, which can last to 2 h after injection (30–32) (**Table 1**). Moreover, in the classical enhancement features of wash-in and wash-out, HCC theoretically appears to be perfusion defects in the Kupffer phase or post-vascular phase because of Kupffer cell shortage (**Figures 1**, **2**). The characteristics of the additional post-vascular phase aid much in HCC detection and diagnosis. Recently, Sonazoid has been proven to be non-inferior to SonoVue in a retrospective clinical study for focal liver lesion (FLL) (33). However, if the lesion is isoechoic in the post-vascular phase, misdiagnosis can happen at a rate of approximately 17% (34). Worse still, owing to histological reasons of some well-differentiated HCC, the sign of perfusion defect in the Kupffer phase could be observed at a rate of only 69% among HCC patients (35). Also, some benign lesions that lack Kupffer cells have a chance to be misdiagnosed as a false-positive sign in the Kupffer phase (36). Therefore, the expected additional clinical benefit on diagnosis gained from the Kupffer phase has not yet been confirmed (37). As for HCC intervention, after US brings real-time monitoring for minimally invasive operations like lesion biopsy and regional ablation, CEUS is employed for more accurate guidance and unique immediate evaluation during therapy (38–43). Vascular-sensitive assessment makes CEUS an indispensable aid for effective radiofrequency (RF)/microwave (MV) ablation (44, 45). On the other hand, three-dimensional (3D) US can provide additional lateral and other viewing angles, and morphological information offers UCA another usable imaging modality (i.e., contrast-enhanced 3D US, CE 3D US) (46, 47) (**Figure 3**). Moreover, contrast enhancement is also employed in fusion imaging to reveal extra small liver lesions and biopsy navigation (48) (**Figure 4**).

### Hepatocyte-Specific Uptake

Mangafodipir trisodium (Mn-DPDP) used to be a classical hepatocyte-selective contrast agent that was developed in the last century and has favorable contrast-to-noise measurements and lesion detection rate as compared to non-enhanced MRI (49, 50). It was high-profile at the beginning for the prolonged enhancement relative to the traditional T1 contrast agents (51). The uptake of Mn-DPDP occurs in hepatocytes, and its elimination is in the biliary tree. Thus, the metabolism process of Mn-DPDP can indicate hepatobiliary function (52, 53). Moreover, it is reported that the hepatocyte-selective contrast agent is correlative with the pathological differentiation degree of HCC (54). Since the uptake of Mn-DPDP strictly occurs in hepatocytes, the extrahepatic originated metastases can be negatively illustrated (55). However, in contrast to the question of how many normal hepatocytes are contained in a lesion, the question of whether a liver lesion is malignant or not will be the highest concern for patients.

By integrating the mechanisms of both hepatocyte-selective contrast agents and non-specific extracellular Gd-chelates, gadolinium-based hepatobiliary-specific agents were thereby developed, such as gadobenate dimeglumine (Gd-BOPTA) and gadoxetic acid (Gd-EOB-DTPA), which are worldwide commercially available and have become a promising MRI contrast agent for FLL (56–58). For HCC diagnostic imaging, the so-called hepatobiliary contrast agents achieve further detection in the early stage for primary, recurrent, and metastatic HCCs through usual dynamic imaging and additional hepatobiliary delayed phase (59–62) (**Figures 1**, **2**). Beyond diagnosis, uptake of Gd-EOB-DTPA of HCC lesions is reported to be a biomarker for prognosis (63), as well as the estimation of liver function (64). Concerning patients’ tolerance, Gd-EOB-DTPA only requires a minimum injection dose to present a satisfying enhancement in the liver and smaller branch of the biliary tree relative to Gd-BOPTA (55) (**Table 1**).

## Molecular Imaging Agents

For the diagnostic and therapeutic purpose of molecular imaging, by means of conjugating some antibody, peptide, or ligand, molecular imaging agents are artificially designed to anchor the targeted cellular and molecular hallmarks pathologically (65).

### Immune Molecular Anchoring

By means of immunoreaction, gadolinium-labeled reagents for liver tumor marking and monitoring of the MR modality are commonly employed in a tumor-bearing animal model for cancer research (66, 67). The molecular weight of reagents mainly ranges from dozens to hundreds of kDa. Likewise, the MBs or nanobubbles binding compounds marked with the tumor-specific immune molecule are also available for cancer research in the CEUS modality (66).

### Stimulus-Responsive/Microenvironment-Dependent Contrast Agents

A T1/T2 switchable MR contrast agent was recently validated on a mouse model for HCC early diagnosis (68, 69). Previously, the diagnostic efficacy of IONP-based MRI was not as high as expected when it was simply employed as a liver-specific T2 agent (70). However, researchers recently found that IONP clusters could be accordingly disaggregated thanks to the acidic tumor microenvironment, which can generate a downstream tumor-specific T1 contrast agent. As a result, the IONP agents can additionally be employed to delineate HCC on T1-weighted images after switching to a downstream tumor-specific contrast agent. Based on IONP, agents decorated with functional small-molecular ligands through surface engineering are thereafter designed to be stimulus-responsive agents, pH-sensitive, and nanoscale distance-dependent (68, 71–75). Furthermore, concerning the aggregation phenomenon that commonly happened in nanoparticles with a large surface area/volume ratio, ultrafine nanoparticles could facilitate intratumoral homogeneous distribution of contrast agents (76). IONP at a diameter of 3.6 nm is supposed to be an optimal T1 agent *in vivo* (77). Moreover, core engineering of various designs of size, shape, composition, surface coating, molecular weight, and drug delivery has indicated IONP to be a hopeful T1 contrast agent (78–85). Beyond imaging, Yang et al. developed a novel nanoparticle that releases Fe^2+^ for the treatment of folic acid (FA) receptor-positive solid tumors through the ferroptosis pathway while being supervised through the Mn agent-enhanced imaging (86, 87). Also, Song et al. developed an assay of therapeutic natural killer cells (NK cells) conjugated with Sonazoid MB to make the antitumor process visible in real-time CEUS (88).

### Scale-Dependent Particles

As nanomedicine was developed recently, emerging nanomaterials have been studied for contrast enhancement imaging. Some nanoscaled CM can permeate into tumor stroma through weak tumor vessels to depict the tumor with or without the assistance from functional parts equipped in advance (89). Moreover, sonoporation induced by external stimulation of focused US can reversibly increase the permeabilization of the cell membrane, leading to the potential visualization of HCC intracellular therapy in the future (90).

## Clinical Challenges and Prospects

As for the clinically commonly used contrast agents, Guang et al. performed a meta-analysis to compare the diagnostic value of CEUS, CT, and MRI in FLL. To rule out HCC from FLL, CECT has the highest sensitivity of 90% (95% CI: 88%–92%), followed by CEUS (88%) and CEMRI (86%). Both CEUS and CEMRI have a higher sensitivity of 81% than CECT (77%). However, all results have no statistical significance (16, 91). Moreover, Westwood et al. found that CEUS could be a cost-effective alternative for HCC diagnosis relative to CECT or CEMRI with similar diagnostic performance (92). Research about combined multimodal medical imaging (including Sonazoid-enhanced US, Gd-EOB-DTPA-enhanced MRI, and CECT) conducted by Masatoshi Kudo figured out that the sensitivity for HCC diagnosis is 72%, 74%, and 86% for CEUS, CECT, and Gd-EOB-DTPA-enhanced MRI, respectively, with no significance among the three imaging modalities. When combining US with MRI, the sensitivity soared as high as 90% (93).

Meanwhile, controversies still remain regarding the diagnostic efficacy of HCC. Despite that the hepatobiliary agent-enhanced MRI is believed to reach an early diagnosis for HCC that is still in the hypovascular stage (94), researchers analyzed the clinical trials that use different contrast agents for HCC diagnosis and found no significant difference in the diagnostic efficacy in terms of sensitivity and specificity between the MRI using extracellular agents and hepatobiliary agents (95, 96). Imbriaco et al. claimed that Gd-EOB-DTPA-enhanced MRI has a better diagnostic performance than CECT only for lesions that are smaller than 20 mm and patients with Child-Pugh class A (97). Moreover, for patients with cirrhosis, Kim et al. demonstrated better performance of hepatobiliary agent-enhanced MRI relative to routine US screening for surveillance of people at a higher risk of HCC (2). In addition, molecular imaging agents, like IONP-based MR agents, are still on the way to fulfilling the various clinical needs (98). On the other hand, although current CM has been deeply improved through materials science, biosafety is still the most crucial factor for patients having various allergies and metabolism troubles. Necessary reinjection of contrast agents for CT and MRI may come with a potential risk of side effects. Minimized dose of contrast agent that meets all clinical needs will be a future trend for CM research.

To sum up, the CM brings out the best diagnostic performance for suitable patients under appropriate conditions. Although Gd-DTPA-enhanced MRI and non-ionic iodinated agents-enhanced CT are usually recommended for HCC diagnosis by mainstream guidelines, liver-specific CM, like Gd-EOB-DTPA and Sonazoid, have already played an anticipated role in HCC diagnosis and prognosis prediction. Furthermore, the amelioration of molecular imaging agents has drawn a blueprint for future medical imaging.

## Author Contributions

Concept and design: KN and YZ. Manuscript writing: YZ. Figure presentation: KN. Reviewed the manuscript: all authors. All authors contributed to the article and approved the submitted version.

## Funding

This work was partially supported by grants from the Natural Science Foundation of Ningbo (No. 2019A610313), Medical Science and Technology Project of Zhejiang Province (No. 2021KY312), Ningbo Medical Science and Technology Project (No. 2019Y05), Ningbo Clinical Medicine Research Center Project (No. 2019A21003), Japan-China Sasakawa Medical Scholarship, and Young Talents Training Program of Ningbo Municipal Health Commission.

## Conflict of Interest

The authors declare that the research was conducted in the absence of any commercial or financial relationships that could be construed as a potential conflict of interest.

## Publisher’s Note

All claims expressed in this article are solely those of the authors and do not necessarily represent those of their affiliated organizations, or those of the publisher, the editors and the reviewers. Any product that may be evaluated in this article, or claim that may be made by its manufacturer, is not guaranteed or endorsed by the publisher.
